# Antioxidant Enzymes Regulate Reactive Oxygen Species during Pod Elongation in *Pisum sativum* and *Brassica chinensis*

**DOI:** 10.1371/journal.pone.0087588

**Published:** 2014-02-04

**Authors:** Nan Liu, Zhifang Lin, Lanlan Guan, Gerald Gaughan, Guizhu Lin

**Affiliations:** 1 Key Laboratory of Vegetation Restoration and Management of Degraded Ecosystems, South China Botanical Garden, Chinese Academy of Sciences, Guangzhou, Guangdong, People’s Republic of China; 2 Department of Biological Sciences, University of Illinois at Chicago, Chicago, Illinois, United States of America; Iwate University, Japan

## Abstract

Previous research has focused on the involvement of reactive oxygen species (ROS) in cell wall loosening and cell extension in plant vegetative growth, but few studies have investigated ROS functions specifically in plant reproductive organs. In this study, ROS levels and antioxidant enzyme activities were assessed in *Pisum sativum* and *Brassica chinensis* pods at five developmental stages. In juvenile pods, the high levels of O_2_^.−^ and^.^OH indicates that they had functions in cell wall loosening and cell elongation. In later developmental stages, high levels of^.^OH were also related to increases in cell wall thickness in lignified tissues. Throughout pod development, most of the O_2_^.−^ was detected on plasma membranes of parenchyma cells and outer epidermis cells of the mesocarp, while most of the H_2_O_2_ was detected on plasma membranes of most cells throughout the mesocarp. This suggests that these sites are presumably the locations of ROS generation. The antioxidant enzymes superoxide dismutase (SOD), peroxidase (POD), and catalase (CAT) apparently contributed to ROS accumulation in pod wall tissues. Furthermore, specifically SOD and POD were found to be associated with pod growth through the regulation of ROS generation and transformation. Throughout pod development, O_2_^.−^ decreases were associated with increased SOD activity, while changes in H_2_O_2_ accumulation were associated with changes in CAT and POD activities. Additionally, high POD activity may contribute to the generation of^.^OH in the early development of pods. It is concluded that the ROS are produced in different sites of plasma membranes with the regulation of antioxidant enzymes, and that substantial ROS generation and accumulation are evident in cell elongation and cell wall loosening in pod wall cells.

## Introduction

**Figure 1:**
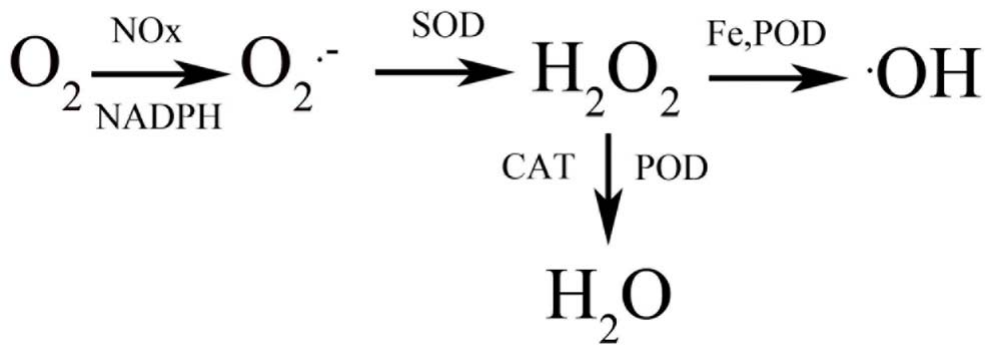
The biosynthesis and degradation of reactive oxygen species (O_2_^.^^−^ **, H_2_O_2_, and^.^OH).** The scheme illustrating reactive oxygen species dynamics in the wall and plasma membrane of plant cells is revised according to [3] and [4]. NOX: NADPH Oxidases.

Oxygen is an important participant in photosynthesis and respiration. To be chemically reactive, dioxygen (O_2_, the common allotrope of elemental oxygen on earth) must be physically or chemically activated [1]. In physical activation, excitation energy from a photo-activated pigment (such as an excited chlorophyll molecule) is transferred to the oxygen molecule to generate singlet oxygen (^1^O_2_). In chemical activation, dioxygen is fully reduced to water. During these processes, intermediates of oxygen reduction that are generated as byproducts include superoxide (O_2_^.−^), hydrogen peroxide (H_2_O_2_), and the hydroxyl radical (^.^OH) [2]. The transformations of these reactive oxygen species (ROS) and their interactions with antioxidant enzymes are illustrated in Figure 1 (revised according to [3], [4]). By interacting with organic molecules such as proteins, carbohydrates, nucleic acids, lipids, and other cellular components, ROS can be toxic and inhibit metabolism and plant growth [5].

ROS can cause phytotoxicity, but they are also known to play an important role in many key plant functions, especially in promoting polysaccharide metabolism, and cell wall loosening and elongation.^.^OH is thought to be involved in the scission of polysaccharides, causing an increase in cell wall extensibility that enables cell growth. [6], [7]. H_2_O_2_ acts as a signal molecule by regulating auxin, which plays a central role in numerous developmental processes that function in cell elongation and differentiation [8]. In growing cells, both O_2_.^−^ and H_2_O_2_ may also directly or indirectly (as a source of^.^OH) promote cell wall softening and cell elongation [9]. In recent years, many studies have focused on the positive functions of ROS in a variety of both plant species and plant organs. Apoplastic ROS have been found to increase cell wall extension in many plants including maize, cucumber, soybean, sunflower, and Scots pine seedlings [4], [7]. ROS have also been documented to have functions in a number of studies concerning plant organs such as roots, embryos, coleoptiles, hypocotyls, leaves and the early seedling growth of different plant species [4], [7], [8], [9], [10], [11], [12].

While there has been much research on ROS involvement in cell wall elongation or extension growth in plants, most previous studies have focused on vegetative tissues, and few studies have investigated ROS functions in the growth and development of reproductive organs. Recently, we have found that ROS are involved in the growth of various flower tissues at early developmental stages of two subtropical woody plants [13]. In this study, we investigated the relationships between ROS and the ontogenesis of *Pisum sativum* and *Brassica chinensis* pods. The pods of these two species make up the main vegetable resources for residents of southern China. The pod growth rate and mature speed of both of these plants are very important for farmers in that they eventually dictate the size of the vegetable during harvest season, but the regulations of these vegetable pods during development are still not well understood. We attempt to answer the following questions: 1) Does ROS accumulation coincide with the cell wall loosening and extension in pod walls? 2) Where are ROS generated in cells of the pod wall? 3) What are the possible functions of ROS and antioxidant enzymes in pod development?

## Materials and Methods

### Pod Samples

No specific permits were required for the field samplings. Seeds of *Pisum sativum* Linn and *Brassica chinensis* Linn. were planted in January 2010 in an experimental field at the South China Botanical Garden, Guangzhou, China. In March 2010, pods of the two species were picked and manually divided into five developmental stages from juvenile to mature based on pod length. The pod lengths of the developmental stages 1 through 5 were 2.2±0.3, 3.0±0.5, 4.1±0.4, 5.0±0.5, and 6.0±0.4 cm for *P. sativum* and 0.8±0.1, 1.1±0.2, 1.8±0.2, 2.1±0.1, and 2.3±0.2 cm for *B. chinensis*, respectively (Table 1).

**Table 1:** Pod wall thickness, pod length, ROS quantitation and activities of SOD, POD, and CAT in pod walls of *P. sativum* and *B. chinensis* throughout the five developmental stages.

Species (pod developmental stage)	Thickness of podwall (mm)	Pod length(cm)	O_2_^.−^ quantitation in the pod walls (pixel ratio of stained area/pod area ×100%)	H_2_O_2_ quantitation in the pod walls (pixel ratio of stained area/pod area ×100%)	SOD activity(units/mg protein)	POD activity (units/min/mg protein)	CAT activity (100×units/min/mg protein)
*P. sativum* (1)	0.380±0.009 a	2.2±0.3 a	84%±11% c	22%±5% b	9.66±0.12 a	2346.58±157.94 d	2.25±0.04 e
*P. sativum* (2)	0.462±0.006 b	3.0±0.5 b	76%±9% c	61%±7% c	10.16±0.10 b	2285.83±74.09 d	1.47±0.14 d
*P. sativum* (3)	0.597±0.012 c	4.1±0.4 c	53%±8% b	58%±6% c	10.65±0.19 c	1104.19±8.44 b	0.59±0.18 c
*P. sativum* (4)	0.774±0.007 d	5.0±0.5 d	52%±6% b	17%±3% b	13.53±0.15 d	1626.82±143.21 c	0.22±0.12 b
*P. sativum* (5)	0.899±0.015 e	6.0±0.4 e	33%±5% a	8%±2% a	15.77±0.40 e	786.82±34.17 a	0.11±0.06 a
*B. chinensis* (1)	0.076±0.004 A	0.8±0.1 A	92%±10% C	11%±2% B	25.81±0.43 B	89.98±2.80 D	1.94±0.35 A
*B. chinensis* (2)	0.109±0.006 B	1.1±0.2 B	88%±8% C	87%±8% D	24.97±0.33 A	83.90±1.11 C	1.91±0.42 A
*B. chinensis* (3)	0.120±0.004 C	1.8±0.2 C	48%±5% B	91%±8% D	28.17±0.15 C	72.81±0.39 B	1.90±0.18 A
*B. chinensis* (4)	0.154±0.007 D	2.1±0.1 D	24%±4% A	25%±6% C	29.69±0.16 D	82.66±3.30 C	1.89±0.23 A
*B. chinensis* (5)	0.189±0.002 E	2.3±0.2 D	22%±4% A	7%±2% A	31.94±0.26 E	56.07±7.90 A	1.90±0.58 A

A sample was collected from both species during each stage of pod development; these samples consisted of five to eight pods in which each pod only collected from one plant. Data are shown as means ± SD. Within each column, a means is given followed by a lowercase letter for *P. sativum* and by an uppercase letter for *B. chinensis*; if the letter is not the same as in the row above, that indicates that the means were statistically different at p<0.05.

### Histochemical Localization of O_2_^.−^ and H_2_O_2_

*In situ* localization of H_2_O_2_ and O_2_.^−^ was performed as described by Romero-Puertas et al. [14]. A sample was collected from both species during each stage of pod development; these samples consisted of five pods, and each pod was collected from a different plant. The pods were infiltrated with 0.5 mg/mL diaminobenzidine (DAB)-phosphate buffer (50 mM, pH 5.8) for histochemical detection of H_2_O_2_ or with 0.5 mg/mL nitroblue tetrazolium (NBT) with 10 mM NaN_3_ in 50 mM HEPES-NaOH buffer (pH 7.6) for histochemical localization of O_2_.^−^. This infiltration was conducted in a vacuum for 30 minutes. The pods were then held at room temperature until the blue color (caused by O_2_^.–^NBT formazan precipitates) or brown color (caused by H_2_O_2_-DAB polymerization) became visible. The chlorophylls contained in the pods were removed by boiling in an ethanol:glycerin (9∶1, v/v) solution to eliminate the background green color in the pods, which had been stained deep-brown or blue. In our previous study on other plant organs, ascorbic acid (a H_2_O_2_ scavenger) and 4,5-dihydroxy-1,3-benzenedisulfonic acid (an O_2_.^−^ scavenger) were used to confirm the effectiveness of ROS histochemical localization methods [5]. Based on our previous results, we believe that O_2_^.–^NBT formazan precipitates and H_2_O_2_-DAB polymerization could well reflect the level of ROS production in pods during development. The stained pods were then photographed with a digital camera and an imaging microscope (DSC-F717, Sony, Japan). The quantitation of H_2_O_2_ and O_2_.^−^ in the pod walls was calculated by the pixel ratio of stained area/pod area ×100%.

### Hydroxyl Radical (^.^OH) Measurement

The concentration of.OH (expressed by relative fluorescence intensity) in pod wall tissues was measured using terephthalic acid (TPA) as a hydroxyl radical dosimeter. A sample was collected from both species during each stage of pod development; these samples consisted of five to eight pods, and each pod came from a different plant. After pod walls were separated from the seeds and homogenized in phosphate buffer (50 mM, pH 7.0), the preparation was centrifuged at 10000×*g* and 4°C for 10 min, and 5 ml of the supernatant ( = pod wall extract) was collected. TPA was used to trap the hydroxyl radicals in the pod wall extracts, and the fluorescent product was measured according to previous studies [15], [16]. The 2-mL total reaction mixtures contained 0.2 mL of 50 µM TPA, 0.2 mL pod wall extract, and 1.6 mL of phosphate buffer (50 mM, pH 7.0). After incubation at room temperature for 6 minutes, the fluorescence emission spectra from 350–550 nm of monohydroxy terephtholate (TPA-OH) was recorded with a fluorescence spectrophotometer (LS 55, Perkin-Elmer, USA) with an excitation wavelength of 326 nm. The.OH production levels in the samples were then calculated and compared based on their maximal emission intensities.

### Microscopic Observation

After the histochemical localization of ROS, samples of the same pod walls that had been subjected to routine fixation and gradient dehydration were infiltrated and embedded with epoxy resin 812. Semi-thin transverse sections (8 µm thick) were prepared with a Leica EM 2016 UC6 microtome, and then the localization of H_2_O_2_ and O_2_.^−^ was observed and photographed with a light microscope (AX70, Olympus, Japan) and a digital camera (DP50, Olympus, Japan). For detecting the structural features of the pod wall, fresh samples from pods at stage 4 were prepared, sectioned, and examined in the same manner except that they were fixed with glutaraldehyde and stained with toluidine blue solution [17].

### Antioxidant Enzyme Activity

A sample was collected from both species during each stage of pod development; these samples consisted of five to eight pods, and each pod came from a different plant. The pod walls at different developing stages were isolated and homogenized in sodium phosphate buffer (50 mM, pH 7.0), and then the supernatant was collected for detection of enzyme activity by centrifugation at 10000×g, 4°C for 10 minutes. Superoxide dimutase (SOD), peroxidase (POD), and catalase (CAT) activities in the supernatant were measured using the following photochemical method. According to Giannopolitis and Ries [18], SOD activity was assayed based on the reduction of NBT (nitroblue tetrazolium). This reaction was conducted in sodium phosphate buffer (50 mM, pH 7.8) containing 100 µl of enzyme extract, 2 µM riboflavin, 65 µM NBT, 13 µM methionine, and 1 µM ethylenediamine tetraacetic sodium. The 3-ml reaction mixture was initialed by illumination for 2 min at 25°C, and the absorbance of blue formazan was measured with a spectrophotometer (UV-3802, Unico, China) at 560 nm. One unit of SOD activity (U) was defined as the amount of enzyme that caused 50% inhibition of NBT reduction.

POD activity was determined by measuring the absorbance changes at 470 nm and 25°C. The reaction was performed in a 3-ml solution. A 10-µl volume of enzyme solution was added to 2.99 ml of sodium phosphate buffer (50 mM, pH 6.0) containing 18.2 mM guaiacol and 4.4 mM H_2_O_2_ as substrates. POD activity was defined as the amount of enzyme that caused an increase in absorbance at 470 nm of 0.001 per minute [19].

CAT activity was measured by monitoring the decrease of H_2_O_2_ at 240 nm for 1 min at 25°C. The 3-ml reaction mixture contained 100 µl of enzyme extract and 2.9 ml of sodium phosphate buffer (50 mM, pH 6.0) containing 10 mM H_2_O_2_. CAT activity was calculated as the amount of enzyme that caused a reduction in absorbance at 240 nm of 0.01 per minute [20].

All of the antioxidant enzyme activity was based on protein concentration; each activity result represents the average of five replications. Protein was determined according to the description by Bradford [21] using bovine serum albumin (BSA) as the standard.

### Statistical Analysis

Results are presented as means ± standard deviation (SD). One-way ANOVAs were applied to compare the antioxidant enzyme activities of pods at different developmental stages. Tukey’s tests were used for *post hoc* multiple comparisons. All the statistical analyses were conducted with SPSS 11.0 (SPSS software Inc., USA).

## Results

### Structure and Thickness of Pod Wall

**Figure 2:**
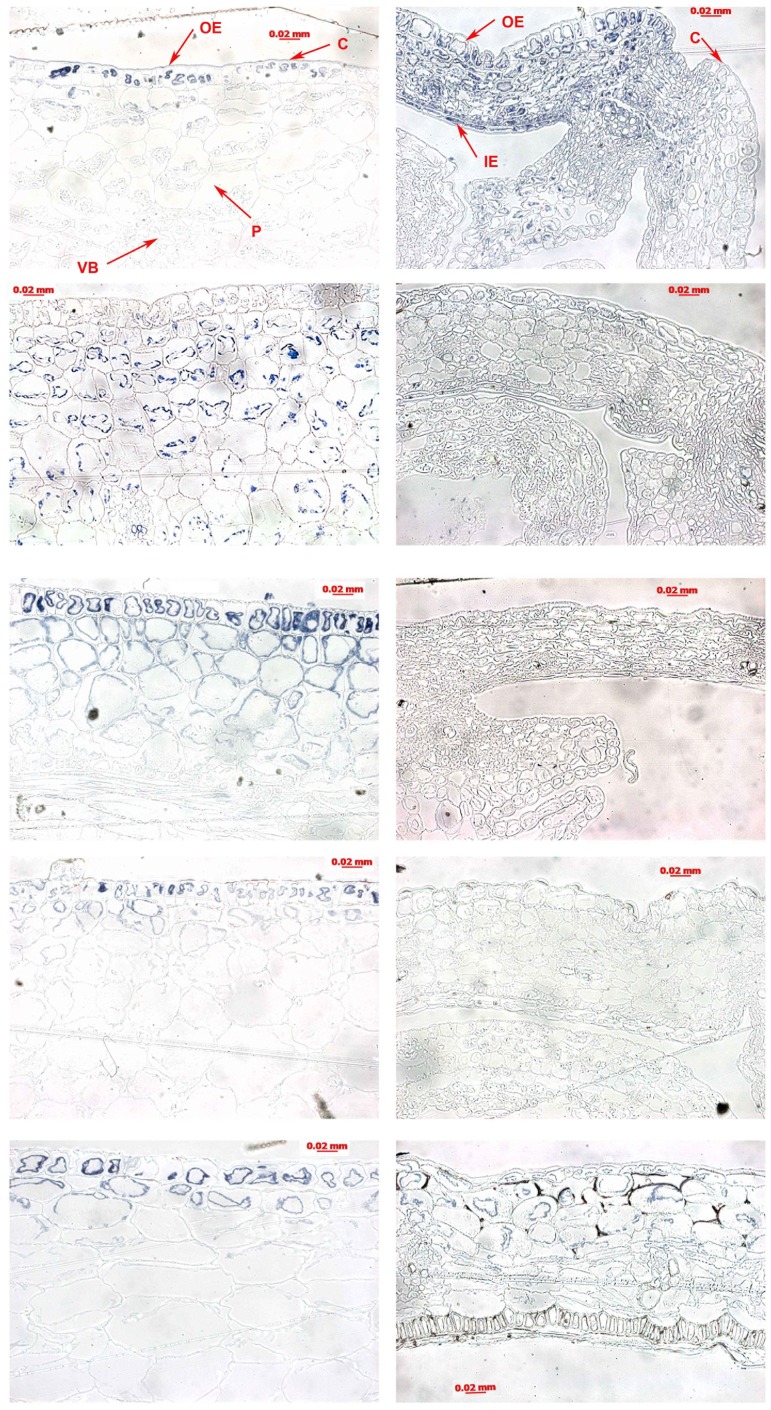
Structural features and thickness of pod walls. Structures of *P. sativum* (left column) and *B. chinensis* (right column) in pods at each developmental stage (from top to down) were detected by light micrographs. Each panel represents microscopic observations of one transverse section of each pod from five replications. A sample was collected from both species during each stage of pod development; these samples consisted of five pods, and each pod was separately collected from a different plant. OE: outer epidermis (exocarp); IE: inner epidermis (endocarp); P: parenchyma (mesocarp); VB: vascular bundle, C: cuticle. Mean pod cell width of *P. sativum* were 0.029±0.008, 0.033±0.011, 0.052±0.015, 0.063±0.021, 0.083±0.030 mm, and of *B. chinensis* were 0.014±0.005, 0.024±0.009, 0.028±0.007, 0.035±0.012, 0.053±0.018 mm.

The pod wall of *P. sativum* consisted of three layers being the exocarp, mesocarp, and endocarp (Fig. 2 left column). The *P. sativum* exocarp consisted of one layer of the outer epidermis and associated stomatal structures. The *P. sativum* mesocarp consisted of some vascular bundles and multiple layers of green parenchyma cells rich in chloroplasts and starch granules. The final layer of the *P. sativum* pod wall, the endocarp, was composed of an inner epidermis and two layers of small parenchyma. The *B. chinensis* pod wall also had three distinct layers but these differed somewhat from those of *P. sativum*. For example, the exocarp of *B. chinensis* included one layer of outer epidermis covered with cuticle (Fig. 2 right column). In addition, the *B. chinensis* mesocarp was constructed of five to six layers of parenchyma cells with vascular bundles and its endocarp was made up of one inner layer of tightly packed, cylindrical sclerenchyma.

For *P. sativum*, the thicknesses of the pod wall were 0.380±0.009, 0.462±0.006, 0.597±0.012, 0.774±0.007, 0.899±0.015 mm during pod development from stages 1 to 5 (Table 1). For *B. chinensis*, the thicknesses were 0.076±0.004, 0.109±0.006, 0.120±0.004, 0.154±0.007, 0.189±0.002 mm from developmental stages 1 to 5. No obvious changes were evident in the shape of the outer and inner epidermis except for the thickening of the cuticle (Fig. 2), suggesting that the mesocarp was responsible for the pod wall thickening associated with pod development.

### H_2_O_2_ and O_2_^.−^ Accumulation in Pod Wall Cells

**Figure 3:**
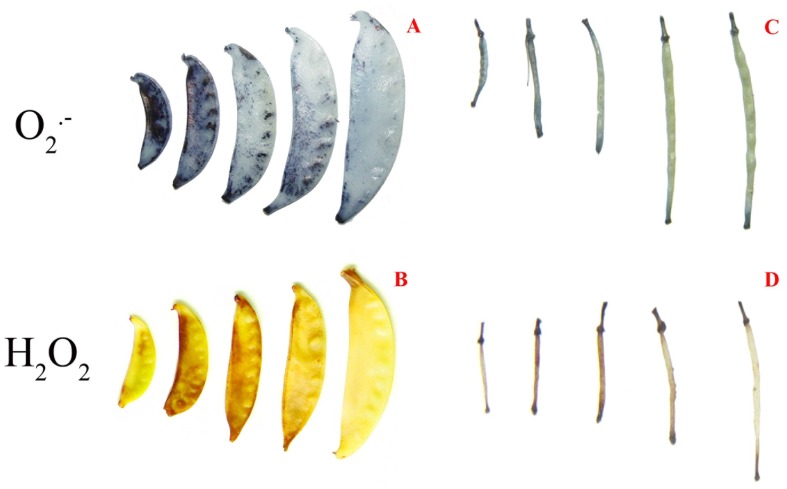
Visualization of reactive oxygen species in pods. O_2_.^−^ (A, C) and H_2_O_2_ (B, D) in pods of *P. sativum* (A,B) and *B. chinensis* (C, D) were detected at five developmental stages. A sample was collected from both species during each stage of pod development; these samples consisted of five pods, and each pod was separately collected from a different plant.O_2_.^−^ and H_2_O_2_ were detected by NBT and DAB staining, respectively.

**Figure 4:**
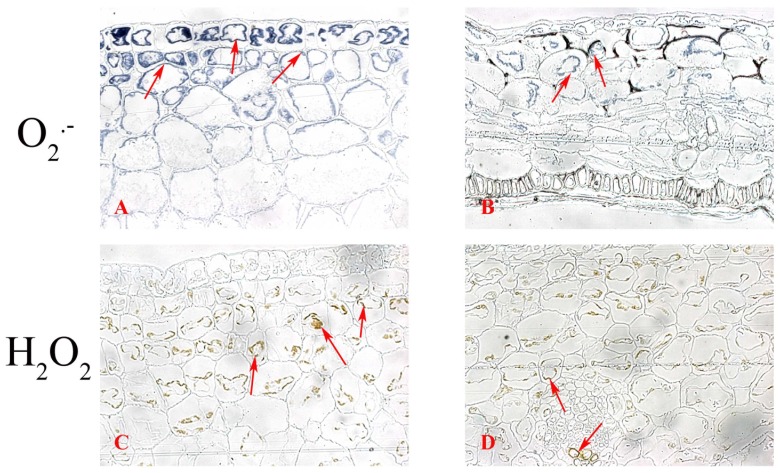
Cytochemical localization of reactive oxygen species in pod walls. O_2_^.−^ (A, B) and H_2_O_2_ (C, D) in transverse sections of pod walls of *P. sativum* (A, B) and *B. chinensis* (B, D) were detected. Each panel represents microscopic observations of one transverse section of each pod from five replications. A sample was collected from both species; these samples consisted of five pods and each pod was separately collected from a different plant. O_2_.^−^ and H_2_O_2_ were detected by NBT and DAB staining, respectively.

Histochemical localization of O_2_.^−^ as indicated by the formation of blue formazan showed that O_2_.^−^ content was high in juvenile pods but decreased as pods of both species matured (Fig. 3). Levels of O_2_.^−^ expressed as a pixel ratio of stained area/pod area were found to be 84%±11%, 76%±9%, 53%±8%, 52%±6% and 33%±5%, with respect to developmental stages 1 through 5, in *P. sativum.* Similar decreasing trends of O_2_.^−^ levels with values of 92%±10%, 88%±8%, 48%±5%, 24%±4% and 22%±4%, respectively, were found for *B. chinensis*. At the cellular level, O_2_.^−^ was mostly located in the outer surface of the *P. sativum* pod wall, especially in the plasma membranes of exocarp epidermis cells and nearby mesocarp cells (Fig. 4A). For *B. chinensis*, in contrast, O_2_.^−^ was equally distributed inside the pod wall cells and was also likely to be present in plasma membranes (Fig. 4B). The homogeneous distribution pattern of O_2_.^−^ in pod wall of *B. chinensis* was mainly due to the thinness of the pod wall, relative to *P. sativum* which was characterized by a small layer of mesocarp cells and of cylindrical shape. The differing patterns of O_2_.^−^ distribution in *P. sativum* were the result of a thick, flat pod wall (4.8-fold the size of *B. chinensis)* which was comprised of numerous chlorenchyma cells in the outer part of pod wall tissue.

Histochemical localization of H_2_O_2_, as indicated by the brown compounds formed by reaction with DAB, first increased and then decreased with development of *P. sativum* pods (Fig. 3B) and *B. chinensis* pods (Fig. 3D). The changes in H_2_O_2_ levels, expressed as a pixel ratio of stained area/pod area were 22%±5%, 61%±7%, 58%±6%, 17%±3% and 8%±2%, with respect to developmental stages 1 through 5, in *P. sativum*. For *B. chinensis* these levels were 11%±2%, 87%±8%, 91%±8%, 25%±6% and 7%±2%, respectively. In contrast to O_2_.^−^, H_2_O_2_ in the pod wall was mostly limited to the inside of the plasma membrane of mesocarp cells and was not evident in the epidermal cells of both species (Fig 4C and D). Brown coloration was also found in the vascular bundle, indicating the possible involvement of H_2_O_2_ in the lignification of vascular cell walls. A negative correlation coefficient (r value) between pod wall thickness or pod length and the O_2_.^−^ level (0.9608–0.9796) or H_2_O_2_ level (0.9008–0.9874) was found in both species (Table 2). These correlations indicate that the longitudinal growth and cross growth of pod walls both requires O_2_.^−^ and H_2_O_2_ generation.

**Table 2:** Correlation coefficients between either pod length or pod wall thickness and either SOD activity, POD activity, CAT activity, or the level of either O_2_^.−^,^.^OH, H_2_O_2_ during pod development.

Species	Item	SOD	POD	CAT	O_2_.^−^	.OH	H_2_O_2_
*P. sativum*	PL	0.9550	−0.8734	−0.9563	−0.9796	−0.1470	−0.9798
	PWT	0.9688	−0.8361	−0.9346	−0.9608	−0.0640	−0.9608
*B. chinensis*	PL	0.9387	−0.7735	−0.8614	−0.9873	−0.5644	−0.9874
	PWT	0.9202	−0.8375	−0.7779	−0.9008	−0.2702	−0.9079

PL, pod length; PWT, pod wall thickness. n = 5–8.

### Hydroxyl Radical (^.^OH) Levels

**Figure 5:**
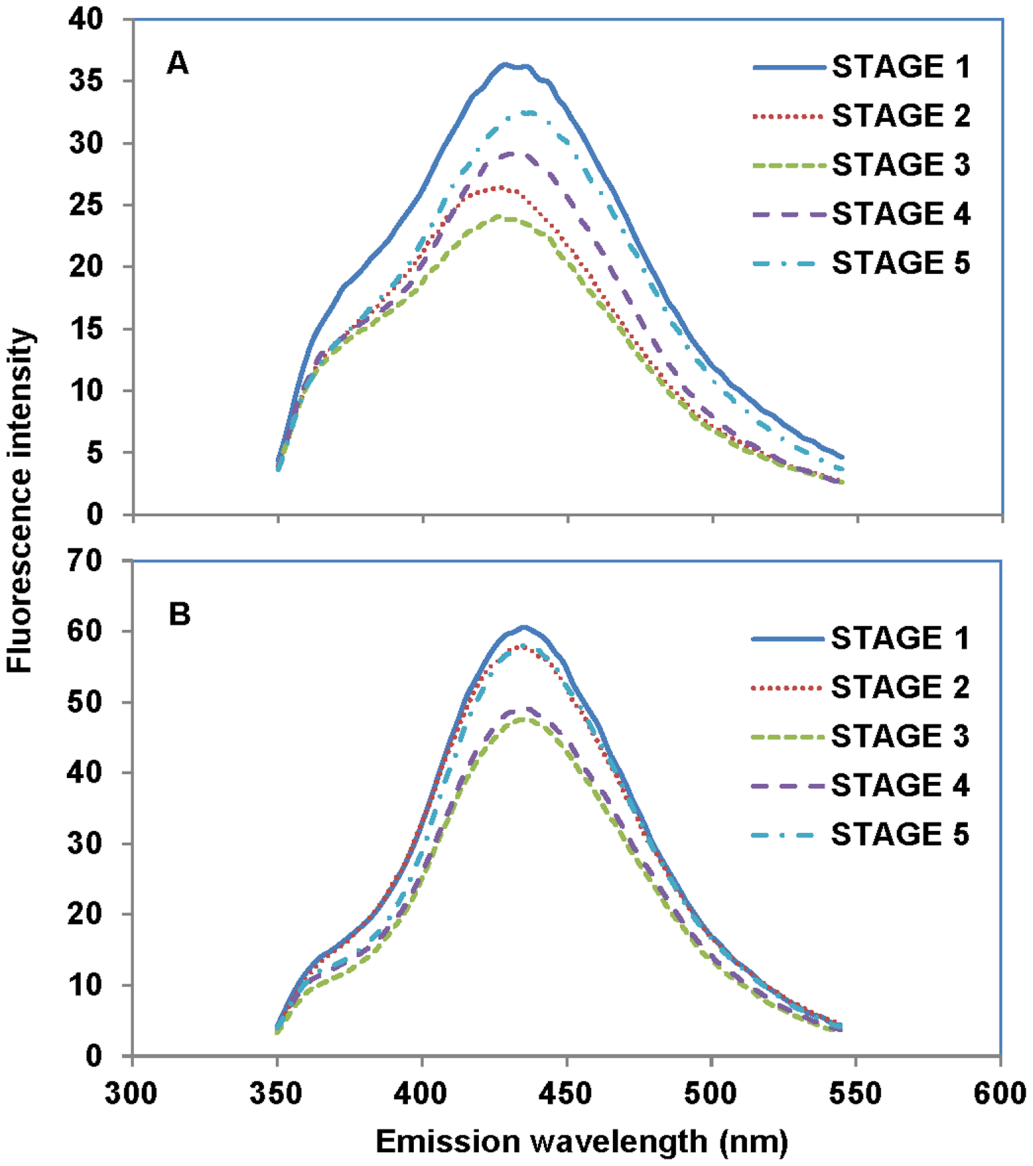
Generation of the hydroxyl radical (^.^OH) in the extracts of the pod walls. ^.^OH of *P. sativum* and *B. chinensis* was detected throughout the five developmental stages. The peak values of *P. sativum* were 36.2±4.0, 26.1±3.4, 23.8±2.1, 29.1±2.7, 32.1±3.0 during pod development from stages 1 to 5. And the peak values of were *B. chinensis* 57.8±4.9, 57.6±4.2, 47.1±3.9, 48.6±2.8, 57.0±3.6 mm from developmental stages 1 to 5. A sample was collected from both species during each stage of pod development; these samples consisted of five to eight pods, and each pod was separately collected from a different plant. Levels of.OH are expressed as relative fluorescence intensity and were measured using terephthalic acid (TPA) as a hydroxyl radical dosimeter. A: *P. sativum*; B: *B. chinensis*.

Fluorescence spectra generated by the TPA-OH assay for pod wall extracts indicated a characteristic peak at 431 nm for both species (Fig. 5). Peaks were lower for *P. sativum* (Fig. 5A) than for *B. chinensis* (Fig. 5B). For *P. sativum*, the peak values were 36.2±4.0, 26.1±3.4, 23.8±2.1, 29.1±2.7, and 32.1±3.0 during pod development from stages 1 to 5. For *B. chinensis*, the peak values were 57.8±4.9, 57.6±4.2, 47.1±3.9, 48.6±2.8, and 57.0±3.6 mm from developmental stages 1 to 5. The fluorescence was highest in juvenile tissue (stage 1) for both species. As pods developed, the fluorescence peak decreased significantly and was lowest in stage 3 and then increased again in stages 4 and 5. Emission peaks were somewhat left shifted in *B. chinensis* pods vs. *P. sativum* pods.

### Antioxidant Enzyme Activity

The activities of SOD, POD, and CAT were measured in pod wall extracts from each of the five developmental stages (Table 1). For both species, SOD activities increased with every developmental stage except that the activity was lower in stage 2 than in stage 1 of the *B. chinensis* pods. SOD activity in pod walls was much greater for *B. chinensis* than for *P. sativum*. In contrast, POD activity was much greater in *P. sativum* than in *B. chinensis*. POD activity for both species tended to decrease with each subsequent pod developmental stage, and the decrease was greater for *P. sativum* than for *B. chinensis*. CAT activity in *P. sativum* pods decreased with pod development, whereas CAT activity in *B. chinensis* pods remained almost unchanged with pod development. In both species, a positive correlation coefficient (r value) was found between pod wall thickness or pod length and SOD activity. There was also a negative correlation coefficient between pod wall thickness or pod length and POD activity. These two correlations show that SOD and POD mediate the ROS metabolism for pod development (Table 2).

## Discussion

There are two main aspects in plant growth: the first is cell division and the second is cell expansion [22]. Both processes allow cells to move from the founder population in and around meristems to the differentiated cells in the mature organ. ROS in plants are important regulators of cell division and expansion, and therefore of plant growth and morphogenesis [23], [24]. The cell division rates, which are roughly estimated by the ratio of pod elongation rates and mean cell widths, were not significantly changed among developmental stages for both species (data not shown). With this in mind, we believe that the increase in pod wall thickness is mainly the result of the enlargement of the cell volume (including the increase of protoplast volume and of the thickness of the secondary wall) in pod wall tissues.

During cell growth, wall thickness is coordinately regulated by biosynthesis and cell expansion [11]. Cell growth depends on the loosening of cell walls and their uptake of water and solutes [25], [26]. Cell wall extension plays a crucial role in plant development, which is promoted by cell wall loosening factors, including expansion protein, hormones, Ca^2+^ influx, ROS and their interactions [27]. In addition to acting as effective cell wall loosening agents, ROS also affect cell growth by activating Ca^2+^ channels [28]. ROS and auxin are important in regulation of plant growth and development. It is revealed that^.^OH mediates the production of auxin, which indirectly contributes in cell extension growth [25]. On the other hand, ROS are capable of being produced in response to auxin, increasing the auxin level triggers H_2_O_2_ production in root tips of tomatos [29]. However, the interactions between the two pathways of auxin and ROS in regulating cell elongation are complex and not well understood. Although SOD, POD, and CAT are components of antioxidant enzyme systems, cell wall-bound SOD and POD participate in the generation of ROS [9]. The generation of ROS required for elongation growth in *P. sativum* has been detected in the cell walls of roots [30], leaves [31], germinated seeds, and seedlings [12] but has not been previously reported from the pods of *P. sativum* or of any other plant species. The generation and localization of ROS as well as the changes in SOD, POD and CAT activities during pod wall development in this study revealed that ROS are an important part of the growth mechanism of the pods. ROS may be a general mechanism for pod wall cell growth, and its role may involve both direct and indirect action. Increasing evidence indicates that^.^OH is a non-specific cell wall loosening agent for the oxidative scission of polysaccharides within cell walls, and that O_2_^.−^ and H_2_O_2_ are the precursors for the formation of^.^OH through either the Fenton reaction or peroxidase-catalyzed reaction in the presence of O_2_ and NADH [26]. As an extremely reactive molecule,^.^OH can attack the cell wall polysaccharide target site, which leads to the breakage of load-bearing structures and allows the wall to stretch [10], [24]. The highest^.^OH level (high^.^OH-TPA fluorescence emission intensity) in pod wall tissue in the current study was detected in stage 1 of pod development, which appears to indicate that^.^OH is required particularly for the early growth of pod wall cells. Our results showed that the shape of pod wall cells was changed from the regular rectangular shape to square or elliptical shape during pod development and that the intercellular spaces were also enlarged throughout the developing stages. This phenomenon when tied in together with the changing rates of ROS generation throughout the developing stages may offer further explanation on the contributions of ROS generation on cell wall loosening throughout pod development.

Plasma membrane-localized NADPH oxidase is responsible for the production of O_2_^.−^ [31]. SOD catalyzes the dismutation of O_2_^.−^ into H_2_O_2_ and O_2._ Therefore, we speculate that the high^.^OH levels in young pod wall detected in this study might result from a high rate of O_2_^.−^ synthesis but a low rate of H_2_O_2_ dismutation, i.e., high POD activity and low SOD activity. The reduced level of O_2_^.−^ in the pod wall at developmental stages 2–4 could be explained by the increased SOD activity and H_2_O_2_ level. In growing cells, O_2_^.−^ and H_2_O_2_ could directly or indirectly promote cell wall softening reactions. In our results, at stage 1, the high level of O_2_^.−^ accumulation was due to the low activity of SOD in transforming O_2_^.−^ into H_2_O_2_. In addition, during stage 1, and O_2_^.−^ may play important roles in cell expansion as a precursor of the^.^OH. Also, H_2_O_2_ accumulation in early to middle developmental stages coincided with increased SOD, and decreased CAT and POD activities, which may infer that H_2_O_2_ acts as a signaling molecule and as a substrate of^.^OH formation for promoting pod wall growth. Thereafter, the decreasing H_2_O_2_ levels together with the gradually decreasing CAT and POD activities presumably led to an increase in^.^OH levels in later developmental stages. We suspect that, in the later stages, more H_2_O_2_ is likely to transform to^.^OH so as to breakdown polysaccharide and mediated the lignification of vascular cell wall in mature pod stage.

ROS generation in plant cell walls during development is a cell/tissue-specific event. ROS appeared on a larger area of pod wall but they predominated in the pod apex. This finding is consistent with previous reports that ROS are mainly present in the young tip zone of growing organs [8], [32]. Microscopic examination of transverse sections of pod walls of both species revealed that O_2_^.−^ and H_2_O_2_ were located in specific sites. Most O_2_^.–^NBT formazan was evident in cells of the outer epidermis and the nearby mesocarp, and most DAB staining of H_2_O_2_ was evident in multiple cell layers of the mesocarp and to a lesser degree in the vascular tissue. These results indicate a significant increase in O_2_^.−^ and H_2_O_2_ generation inside the expanding pod wall. As noted earlier, O_2_^.−^ is generated by plasma membrane-bound NADPH oxidase using NADPH as an electron donor, and the dismutation of O_2_^.−^ to H_2_O_2_ is catalyzed by cell wall SOD [4]. Therefore, the presence of O_2_^.−^ and H_2_O_2_ in plasma membranes of pod cell walls is consistent with a prominent role of NADPH oxidase and SOD. Confirming these inferences will require additional research.

## Conclusion

Taken together, our results provide a starting point for understanding the involvement of ROS in pod growth. We conclude that:

1. Substantial levels of ROS are generated in the cells of pod walls during pod development. The changes in^.^OH, O_2_^.−^, and H_2_O_2_ levels during pod wall development may be due to possible transformations among these three different ROS.
2. The larger quantity of both O_2_^.−^ and^.^OH in pod walls of both species in early vs. later developmental stages suggests that they have important roles in cell elongation and cell wall loosening of the pod wall cells. Whereas in mature pods, the high^.^OH level in the pod wall suggests its possible function in increasing the thickness of cell walls of lignifying tissues.
3. During pod development, O_2_^.−^ generation was mainly detected on the plasma membranes of outer epidermis cells and inner parenchyma cells of the mesocarp. Meanwhile, H_2_O_2_ was mainly detected on the plasma membranes of most mesocarp cells and to a lesser degree on the plasma membranes of vascular cells. These detection sites are presumably the sites of ROS generation.
4. Antioxidant enzymes are evidently involved in the transformation among different ROS in pods walls. The positive relation between pod wall thickness and SOD activity and the negative relation between pod wall thickness and POD activity may suggest that these two enzymes are responsible for pod growth through the regulation of ROS generation. The decrease in O_2_^.−^ levels in pod walls was associated with an increase in SOD activity as pods developed. The initial decrease and subsequent increase in H_2_O_2_ levels were mainly associated with changes in CAT and POD activities during pod development. Moreover, the high activity of POD is also likely to participate in the formation of^.^OH in early pod development.
